# Forecasting Mood in Bipolar Disorder From Smartphone Self-assessments: Hierarchical Bayesian Approach

**DOI:** 10.2196/15028

**Published:** 2020-04-01

**Authors:** Jonas Busk, Maria Faurholt-Jepsen, Mads Frost, Jakob E Bardram, Lars Vedel Kessing, Ole Winther

**Affiliations:** 1 Department of Applied Mathematics and Computer Science Technical University of Denmark Lyngby Denmark; 2 Copenhagen Affective Disorder Research Center Psychiatric Center Copenhagen Rigshospitalet Copenhagen Denmark; 3 Monsenso ApS Copenhagen Denmark; 4 Department of Health Technology Technical University of Denmark Lyngby Denmark; 5 Faculty of Health and Medical Sciences University of Copenhagen Copenhagen Denmark; 6 Center for Genomic Medicine Rigshospitalet, Copenhagen University Hospital Copenhagen Denmark; 7 Bioinformatics Centre Department of Biology University of Copenhagen Copenhagen Denmark

**Keywords:** bipolar disorder, mood, early medical intervention, digital phenotyping, machine learning, forecasting, Bayesian analysis

## Abstract

**Background:**

Bipolar disorder is a prevalent mental health condition that is imposing significant burden on society. Accurate forecasting of symptom scores can be used to improve disease monitoring, enable early intervention, and eventually help prevent costly hospitalizations. Although several studies have examined the use of smartphone data to detect mood, only few studies deal with forecasting mood for one or more days.

**Objective:**

This study aimed to examine the feasibility of forecasting daily subjective mood scores based on daily self-assessments collected from patients with bipolar disorder via a smartphone-based system in a randomized clinical trial.

**Methods:**

We applied hierarchical Bayesian regression models, a multi-task learning method, to account for individual differences and forecast mood for up to seven days based on 15,975 smartphone self-assessments from 84 patients with bipolar disorder participating in a randomized clinical trial. We reported the results of two time-series cross-validation 1-day forecast experiments corresponding to two different real-world scenarios and compared the outcomes with commonly used baseline methods. We then applied the best model to evaluate a 7-day forecast.

**Results:**

The best performing model used a history of 4 days of self-assessment to predict future mood scores with historical mood being the most important predictor variable. The proposed hierarchical Bayesian regression model outperformed pooled and separate models in a 1-day forecast time-series cross-validation experiment and achieved the predicted metrics, *R^2^*=0.51 and root mean squared error of 0.32, for mood scores on a scale of −3 to 3. When increasing the forecast horizon, forecast errors also increased and the forecast regressed toward the mean of data distribution.

**Conclusions:**

Our proposed method can forecast mood for several days with low error compared with common baseline methods. The applicability of a mood forecast in the clinical treatment of bipolar disorder has also been discussed.

## Introduction

### Background

Bipolar disorder is estimated as one of the most important causes of disability worldwide [1,2]. Bipolar disorder is characterized by recurrent episodes of depression, (hypo)mania, and mixed episodes intervened by periods of euthymia [3] and with a high degree of comorbidity, functional impairment, and increased risk of suicide [4]. The World Health Organization estimates that about 60 million people are affected by bipolar disorder worldwide and that the burden of depression and other mental health conditions is on the rise globally [5]. The cornerstone of treatment of patients with bipolar disorder is continuous and long-term maintenance treatment to reduce or prevent relapses, applying a variety of methods including psychopharmacological treatment and group-based psychoeducation. Long-term treatment also involves symptom monitoring, early identification of subsyndromal symptoms of depression and mania, and intervention to prevent or reduce the severity of affective episodes.

In this paper, we analyzed daily self-assessments, including mood scores, collected from patients with bipolar disorder through a smartphone-based system. Ecological momentary assessment (EMA) reflects the method used to collect assessments of individuals’ real-time states repeatedly, over time, during naturalistic settings and may reduce recall bias [6]. At present, a median of 76% of adults across 18 advanced economies reported having a smartphone [7], and many people use a smartphone daily [8]. The rapid evolution and ubiquity of mobile networks have resulted in the increasing growth of electronic mental health technologies, including electronic platforms offering tolls for remote self-monitoring [9]. By using daily smartphone-based self-monitoring, potential recall bias in self-reported patient data is minimized. Thus, smartphones extend the use of EMA beyond its classical use for self-reports and offer the opportunity to collect fine-grained data unobtrusively and outside clinical settings [10]. By replacing paper-based self-assessments of traditional treatment methods with a smartphone-based system, users can ubiquitously enter and review their own data and share the data with clinicians, who can intervene if something appears alarming. Thus, smartphones provide a unique platform for monitoring and managing depression and mania [11-13]. Furthermore, modern smartphones provide the means for collecting rich sensor data, which are believed to capture valuable behavioral information that can be related to disease outcomes [14]. Digital self-reporting and data collection have the additional benefit of making data available for automatic analysis immediately, which can help support continuous disease monitoring.

We found it useful to distinguish between *mood detection*, ie, predicting the mood based on data from the same day, and *mood forecasting*, ie, predicting the mood one or more days ahead based on historical data. Smartphone-based mood detection is well studied but remains a difficult problem. Several papers have examined the use of passive smartphone data, such as location, communication logs, and device usage, to detect or classify daily self-reported mood labels [15-21]. A few recent studies [22,23] have addressed mood forecasting, which is a more challenging task than mood detection, as the causal chain between cause and outcome is longer and because of the uncertainty inherently associated with future events. DeepMood by Suhara et al [22] is a solution for forecasting severely depressed mood from self-reported histories using a recurrent neural network. Suhara et al [22] found that long-term historical information up to 14 days improves the accuracy of forecasting depressed mood classes and that the mood on the previous day is the most important predictor when forecasting severe depression for one day. A limitation of this method is that it needs labeled observations every day in a 14-day history to make predictions. A study by Taylor et al [23] employed a selection of multi-task learning (MTL) techniques to train personalized models for predicting future mood, stress, and health one day ahead. Taylor et al [23] found that using MTL techniques to account for individual differences provides substantial performance improvements over traditional machine learning methods. By utilizing a cluster of users based on age, gender, and personality, a new user needs only to be assigned to a cluster to enable prediction based on new data, when labeled data from a population of similar users are available to fit the initial model.

A major challenge when reviewing work on mood prediction and behavior tracking is that researchers often have different data collection strategies and apply custom modelling and labeling approaches, consequently making results difficult to compare and sometimes contradicting [14]. Another limitation is that most studies involve healthy subjects (ie, without a diagnosis), and it is therefore hard to generalize to patients suffering from affective disorders. Nonetheless, some common observations stand out. Several studies found that personalized models generally outperform generic models when sufficient data are available [16,19,23-25], demonstrating the importance of accounting for individual differences in the data. This can be accommodated by applying MTL techniques, which provides a way of improving generalization by learning several related tasks simultaneously [26]. By considering individuals as separate tasks in a combined model, MTL techniques can produce personalized predictions in a straightforward manner.

Our study differs from prior work in a number of ways. Where many studies collect data from nonclinical subjects (such as students and volunteers recruited on the Web), our data are collected in a randomized clinical trial from patients who received a diagnosis of bipolar disorder and were treated for it. Moreover, to the best of our knowledge, the size of our patient population (*N*=84) is significantly larger than any prior clinical studies. We also found that even though most studies record subjective mood on a continuous or ordinal scale, the prediction task is often reduced to a classification problem by binning the values into two or more classes, such as *neutral*, *depressed*, and *manic*. In this study, we treated mood prediction as a regression problem, which is more direct given the way data are collected and interpreted by users. Finally, rather than mood detection, we addressed the more challenging task of mood forecasting and applied a hierarchical Bayesian modelling approach, which is a popular method of MTL that is able to account for individual differences in the data.

### Objectives

The main objective of this study was to examine the feasibility and technical foundation of forecasting daily mood scores in bipolar disorder based on daily smartphone self-assessments. We hypothesized that utilizing these data to establish an accurate, real-time mood forecast solution can help improve disease monitoring by providing additional insights that enable early intervention and thus eventually prevent the relapse of affective episodes and burdensome and costly hospitalizations.

## Methods

### Data Description

Data used in this study were collected between September 2014 and January 2018 during the MONARCA II randomized clinical trial [27] that was investigating the effect of smartphone-based monitoring. All patients with a diagnosis of bipolar disorder who had previously been treated at the Copenhagen Clinic for Affective Disorder, Copenhagen, Denmark, in the period from 2004 to 2016 and who, at the time of recruitment, were being treated at community psychiatric centers, by private psychiatrists, and by general practitioners were invited to participate in the trial. Patients were included in the study for a 9-month follow-up period if they received a diagnosis of bipolar disorder according to International Classification of Diseases, 10th Revision using the Schedules for Clinical Assessments in Neuropsychiatry [28] and were previously treated at the Copenhagen Clinic for Affective Disorder. Patients with schizophrenia, schizotypal, or delusional disorders, previous use of the MONARCA system, pregnancy, and a lack of Danish language skills were excluded. Patients with other comorbid psychiatric disorders and substance use were eligible for the trial. As a part of the MONARCA II trial, patients were randomized to either using a smartphone-based monitoring system (the Monsenso system) for daily self-monitoring (the intervention group) or treatment as usual (the control group). Patients included in the intervention group collected daily smartphone-based self-monitoring data and were included in the analyses in this paper. The inclusion and exclusion criteria were investigated and assessed by 1 clinical researcher (MJ).

Study participants were provided an Android smartphone app configured for the study and were instructed to evaluate subjective measures of illness activity on a daily basis by answering a daily self-assessment questionnaire including the items listed in Table 1. Specifically, mood was scored on a scale of −3, −2, −1, −0.5, 0, 0.5, 1, 2, and 3, where negative values indicate various degrees of depression, positive values indicate mania, and zero indicates neutral mood (euthymia).

**Table 1:** Items of the daily self-assessment questionnaire.

Attribute	Description	Value
Activity	Level of physical activity	−3 to 3
Alcohol	Alcoholic drinks consumed	0 to 10+
Anxiety	Level of anxiety	0 to 2
Irritable	Level of irritability	0 to 2
Cognitive difficulty	Level of cognitive discomfort	0 to 2
Medicine	Medicine adherence	0 to 2
Mixed mood	Experienced mixed mood	0 to 1
Mood	Experienced mood	−3 to 3
Sleep	Hours of sleep	0 to 24
Stress	Level of stress	0 to 2

Additionally, study participants were periodically evaluated by trained psychiatrists throughout the trial, up to five times (at baseline and after 4 weeks, 3 months, 6 months, and 9 months), on the following clinical rating scales for depression and mania: the Hamilton Depression Rating Scale (HDRS) [29] and Young Mania Rating Scale (YMRS) [30]. Each rating scale consists of a series of questions that are scored and totaled to summarize the current state of the patient with higher scores indicating more severe symptoms. Clinical researchers, who were blinded to all smartphone-based data, conducted all the clinical assessments. Thus, data on the severity of depressive and manic symptoms were collected in a rater-blinded manner. Both rating scales are clinically validated and generally accepted as accurate measures of illness severity in bipolar disorder.

### Data Preprocessing

Two of the self-assessment items were preprocessed before the analysis. As the answer to the medicine item is categorical by design (medicine not taken, medicine taken, and medicine taken with changes), we encoded it as two exclusive binary variables indicating if medicine was not taken (medicine omitted) or if medicine was taken with changes (medicine changed). Additionally, we did not expect sleep duration to have a linear effect on mood, thus the sleep variable was replaced with two new features by subtracting the individual mean and splitting the result into a negative and positive component (sleep negative and sleep positive), indicating decreased or increased sleep relative to the mean. Finally, we normalized all self-assessment variables by their allowed minimum and maximum value. We also experimented with forward filling the missing data from the previous day but found that very little additional data were gained; therefore, we left this step out of the final analysis presented in this paper.

### Forecasting

**Figure 1:**
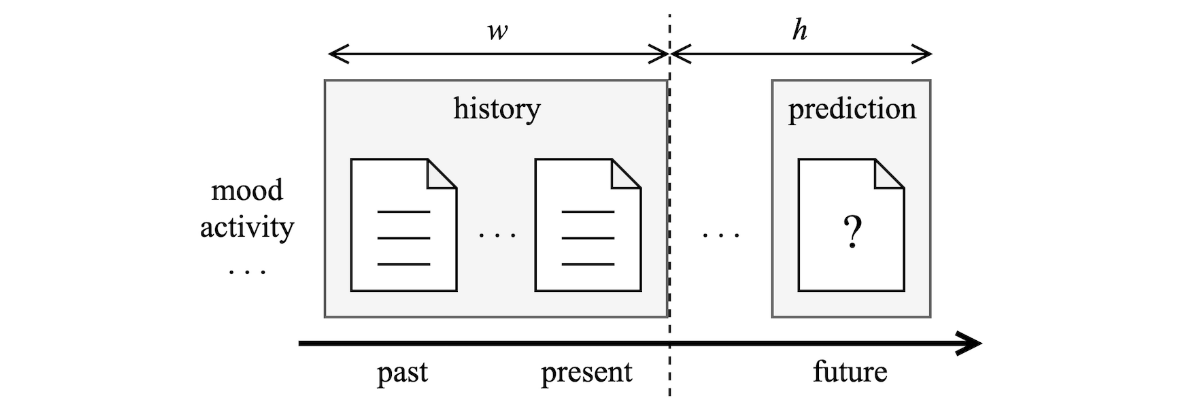
Forecasting is the task of predicting the future, given all relevant information from the past and the present. The window size, w, is the size of history defining the predictor variables and the horizon, h, is how far in the future the target variable is predicted.

Forecasting is the task of predicting the future, given all available information from the past and present [31,32]. For forecasting to be feasible, it should be reasonable to assume that the history of recorded information somehow relates to the predicted future events. A typical forecasting task is illustrated in Figure 1; *w* denotes the size of history used in the forecast and *h* denotes the horizon of how far into the future the target is predicted. In our case of using daily self-reports, both *w* and *h* are measured in days.

Several methods for producing forecasts exist [32]. The simplest forecasting methods use only historic information about the target variable and do not consider any other information but are designed to utilize time-dependent patterns, such as seasonality and trend, to extrapolate observed data into the future. Another approach is to apply standard regression or classification models to predict the variable of interest based on relevant information, such as prior (lagged) observations of the target variable along with additional predictor variables. This approach has the benefit of allowing the use of a variety of different methods from the machine learning and statistical inference literature but may not be as good at capturing long-term time-dependent patterns. For short-term forecasts or data without long-term time dependence, however, this might not be a problem. For these reasons, we chose to apply the latter approach in this work.

Special care should be taken when evaluating the performance of a forecast. A genuine forecast only uses data available at the time of forecast, and thus no future data, to estimate its parameters [32]. Consequently, the size of in-sample residuals (training error) is not a reliable indicator of the true forecast error. The forecast performance can only be determined by fitting the model on training data observed before the test data. This needs to be considered when designing the experiment used to evaluate the forecast model, such as cross-validation. Time-series cross-validation addresses this by splitting the data into a sequence of consecutive test sets. The corresponding training sets consist only of data observed before each test set. Thus, no future information is included when constructing the forecasts. The cross-validation error is then computed across all the test sets. As we considered data from multiple individuals, we applied two different time-series cross-validation in our experiments:

1. Leave-all-out time-series cross-validation: Each individual’s data are partitioned into a sequence of *T* consecutive similar-sized test sets. Then the test sets are pooled across all individuals. The corresponding training sets consist of all data observed before each test set, resulting in *T*−1 test and training set pairs (the first test set has no prior data).
2. Leave-one-out time-series cross-validation: Each individual’s data are partitioned into a training set and subsequent test set. The training set is then pooled with all data from all other individuals, resulting in a number of test/training set pairs equal to the number of individuals, *J*.

These two experiments correspond to two different scenarios: the leave-all-out time-series cross-validation simulates a situation where a group of patients starts monitoring at the same time without any additional historical data, whereas the leave-one-out time-series cross-validation simulates a situation where each participant starts monitoring when data are already available from a population of similar individuals.

### Hierarchical Bayesian Models

When analyzing data consisting of multiple related sets of measurements, such as individuals in a population, a basic approach is to completely pool all the data into a common model, assuming all sets have similar properties. A drawback of this method is there is a risk of losing important information at the individual level. To overcome this problem, an alternative approach is to model each set of data separately, assuming all sets are independent. However, information about how the individual sets relate to each other at the population level might be missed. Especially when each individual dataset is too small to construct a meaningful separate model, it is useful to include information from the population to make analysis feasible. A hierarchical (multi-level) Bayesian model is an intermediate solution allowing partial pooling of the data, thus providing a compromise between the completely pooled and separate models [33,34]. The hierarchical approach captures the overall characteristics of the population while allowing individual differences and enables modeling of small related datasets, each getting a gradually more personalized model as more data are collected and included in the training set. Additionally, it allows for reasoning about previously unobserved individuals, assuming they come from the same population, which helps to overcome the cold start problem. Applying a Bayesian approach has the additional benefit of providing uncertainty in all model parameters and predictions, allowing for improved interpretability. Owing to these desirable properties, we applied hierarchical models in our analysis. In particular, we explored the use of hierarchical implementations of both linear and ordinal regression models.

**Figure 2:**
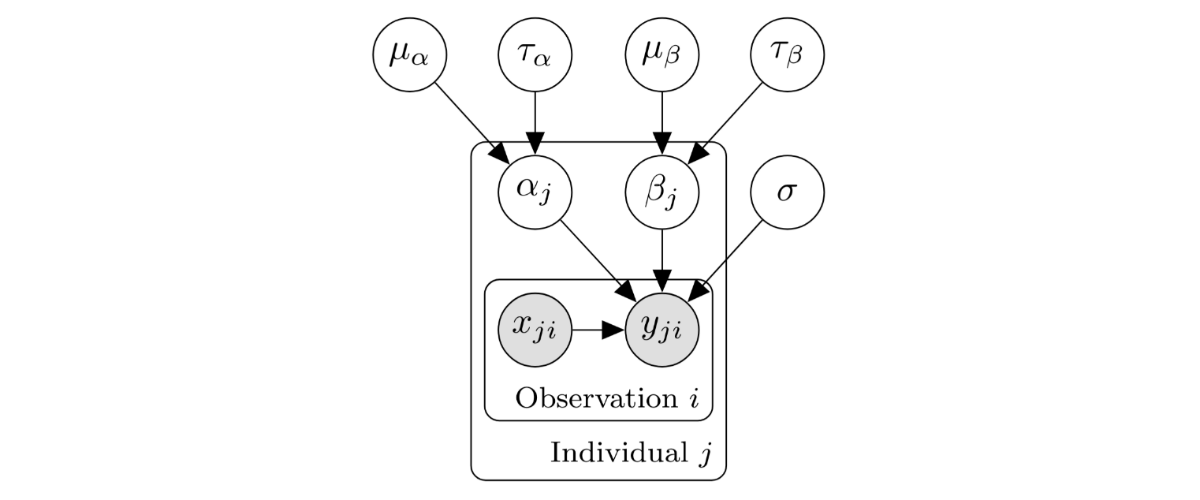
A Bayesian network of a hierarchical linear regression model. Individual regression intercept α_j_ and weights β_j_ are drawn from population distributions parameterized by μ_α_, τ_α_ and μ_β_, τ_β_. This allows the model to account for individual differences while constraining individual parameters to be similar across the population.

Ordinary linear regression is a method of predicting the outcome of a continuous variable, modeled as the linear combination of the model parameters and predictor variables. Hierarchical Bayesian linear regression can be expressed by assuming that each set of parameters is drawn from a common population distribution (Figure 2). For individual *j*=1:*J*, observation *i*=1:*N*, target variable *y_ji_*, and predictor variables ***x_ji_***:

*y_ji_*=Normal(*α_j_* + *β_j_^T^
**x_ji_***,*σ*)

where *α_j_* and *β_j_* are sampled from population distributions:

*α_j_*~Normal(*μ_α_*_,_*τ_α_*)

*β_j_*~Normal(*μ_β_*_,_*τ_β_*)

and the population means *μ_α_*, *μ_β_* and variances *τ_α_*_,_ *τ_β_* as well as the standard error *σ* have independent normal priors.

Ordinal regression (sometimes referred to as ordinal classification) is a method of predicting a discrete variable that has a relative ordering of the possible outcomes. Thus, it can be thought of as an intermediate between regression and classification. An example of ordinal regression is ordered logistic regression. For an outcome belonging to one of *K* categories, y*_ji_* ∈ 1:*K*, ordered logistic regression is determined by a latent continuous variable, *z_ji_*=*β_j_^T^ **x_ji_***, along with a sequence of *K*+1 ordered cutpoints, ***c_j_***, such that *c_k_*_−1_<*c_k_* and *c*_0_=−∞, *c*_K_=∞ by definition. If *z_ji_* falls between two cutpoints, *c_k_*_−1_ and *c_k_*, the outcome is predicted to belong to the corresponding category, y*_ji_*=*k*, with high probability. This type of model can be justified by assuming the category, y*_ji_*, is an incomplete measurement of the latent variable, *z_ji_*:

*y_ji_*~OrderedLogistic (*z_ji_*, ***c_j_***)

Hierarchical Bayesian ordinal regression can be expressed by assuming that each set of model parameters is drawn from a common population distribution:

*β_j_*~Normal (*μ_β,_ τ_β_*)

***c_j_***~Normal (*μ_c,_ τ_c_*)

with independent normal priors on the population parameters, *μ_β,_ τ_β,_* *μ_c,_ τ_c_*, along with ordering constraints on *μ_c_* and ***c_j_***. In practice, we re-parameterized the hierarchical models to achieve more efficient sampling [35]. A practical difference of using ordinal regression over linear regression is that ordinal regression can never produce predictions (or uncertainties) outside the range of the training data. This can be an advantage when the target variable represents a bounded scale where values outside the scale do not have any meaning. Ordinary linear regression does not provide this guarantee; thus, the ordinal model can lead to more interpretable outcomes.

We used the open-source statistical modeling platform, Stan [36], to specify and perform inference in the hierarchical models. Generally, the models were fitted using four sampling chains and 5,000 iterations, where the first half was warm-up and parameter tuning, resulting in 10,000 posterior samples. Our prior belief was that self-reported mood would be the strongest predictor of future mood, hence the population parameters corresponding to mood were assigned less restrictive priors than the other population parameters, which were assigned more restrictive priors to introduce regularization. The Stan code of the hierarchical models and more details on the priors is included in Multimedia Appendix 1. To provide appropriate baseline results for comparison, a suite of naïve and standard machine learning regression models from the Scikit-learn machine learning library [37] and the popular XGBoost Python package [38] were also evaluated. These models were fitted both with pooled and separate data, where applicable.

### Ethical Considerations

The Regional Ethics Committee in the Capital Region of Denmark (H-2-2014-059) and the Danish Data protection agency (2013-41-1710) approved the trial. The law on handling of personal data was respected. Before commencement, the trial was registered at ClinicalTrials.gov (NCT02221336). Electronic data collected from the smartphones were stored at a secure server at Concern IT, Capital Region, Denmark (I-suite number RHP-292 2011-03). The trial complied with the Helsinki Declaration of 1975, as revised in 2008.

## Results

### Descriptive Statistics

**Figure 3:**
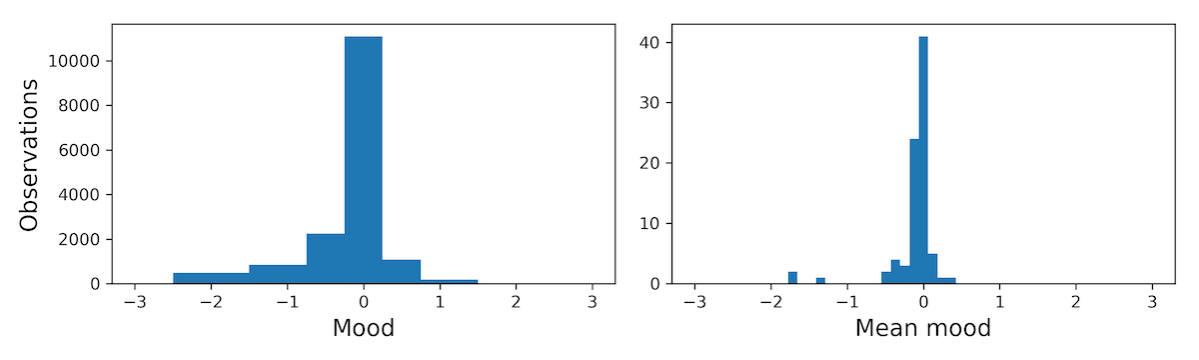
Distribution of all self-reported mood scores (left) and individual mean mood scores (right). The mood scores are generally close to zero indicating neutral mood with only a few exceptions indicating depressed or elevated mood.

The dataset consists of 15,975 daily self-assessments and 280 clinical evaluations from 84 participants. This corresponds to an average of 190.2 self-assessments per individual and an average self-assessment adherence of 82.8% between the first and last submitted self-assessment. The population ranged from the ages of 21 to 71 years (mean 43.1, SD 12.4) and consisted of 62% (52/84) women. Figure 3 presents the distribution of self-reported mood scores across all individuals in the dataset (mean −0.14, SD 0.48). The majority of observed mood scores, *y*, are centered around zero, indicating euthymia (−0.75<*y*<0.75=89.64%) with only few values indicating depression (*y*<−0.75=8.68%) and even fewer values indicating mania (*y*>0.75=1.68%). As expected, the self-reported mood scores and HDRS scores were negatively correlated (*r*=−0.40; *P*<.001) and self-reported mood scores and YMRS scores were positively correlated (*r*=0.22; *P*<.001).

**Figure 4:**
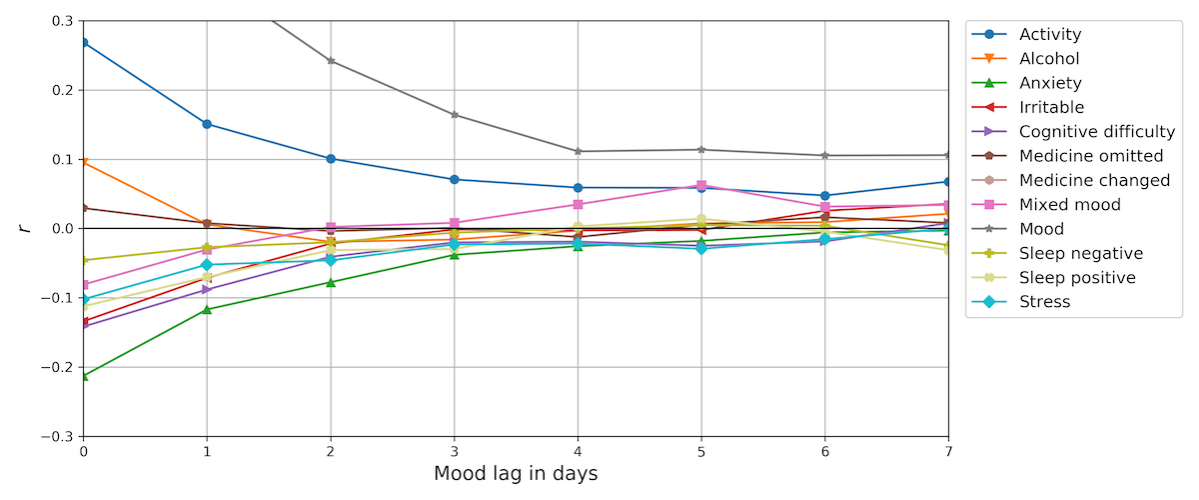
The mean of individual correlations of self-assessment items and mood lagged up to 7 days. Nonzero correlations indicate that items have some relation to mood on subsequent days that can be utilized for mood forecasting.

Figure 4 shows correlations of self-assessment items with self-reported mood lagged for up to seven days. Self-reported mood has a positive autocorrelation for the entire duration of 1 to 7 days. Additionally, activity has a positive correlation with mood for a few days, indicating that high activity levels coincides with elevated mood, and anxiety has a small negative correlation with mood, indicating that anxiety often coincides with negative mood scores. The remaining self-assessment items show small, diminishing correlations with lagged mood. A seasonality analysis of self-reported mood revealed no significant monthly or daily seasonality in the data and was left out for brevity.

### Window Size Selection

To find the optimal window size, *w*, for forecasting mood, we evaluated a 1-day forecast with window sizes from 1 to 7 days. Each window size was evaluated in a *T*=24 leave-all-out time-series cross-validation experiment with data partitions with a size of one week. The predicted coefficient of determination (*R*^2^), indicating the proportion of the data variance explained by each model (higher is better), and the root mean squared error (RMSE), measuring the square root of the mean of squared errors (lower is better), were computed across all the test sets.

**Figure 5:**
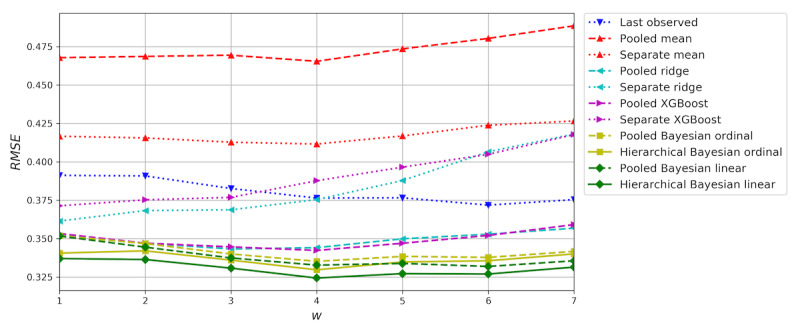
Window size selection results. The root mean squared error (RMSE) was evaluated in time-series cross-validation experiments for w=1 through 7 and h=1. The lowest RMSE was achieved by the hierarchical linear model at w=4.

Figure 5 shows the RMSE of the cross-validation for *w*=1 through 7 and *h*=1. The errors of the naïve mean models are almost constant, varying only because of the difference in datasets available for different values of *w*. The hierarchical Bayesian linear regression model achieved the lowest RMSE of all models for every window size with the best result at *w*=4 days, which we then used in the following analysis.

### Model Checks and Feature Importance

To evaluate how well the proposed hierarchical linear and ordinal models fit the data distribution, we trained them on the entire dataset of participants with at least two data points for *w*=4 and *h*=1 (*N*=5881). The hierarchical models achieved a similar fit with in-sample *R*^2^=0.56 and in-sample RMSE=0.29. We then performed posterior predictive checks by testing the ability of the models to replicate (predict) the observed distribution of future mood from the observed history of predictor variables. In particular, we computed the ratio of observed mood values and replicated mood values less than −0.75 and greater than 0.75. The hierarchical linear model replicated 93% of the small values while the ordinal model replicated 65% of the small values. The hierarchical linear model replicated 73% of the large values while the ordinal model only replicated 24% of the large values. Thus, the hierarchical linear model is better at capturing the tails of the distribution whereas the ordinal model underestimates extreme values.

The importance of a predictor variable in a linear regression model can be measured as the absolute value of the *t*-statistic of its regression weight, *β*, computed as the mean weight scaled by its standard error: *t_β_*=*β/SE(β)* [39]. Table 2 presents the mean absolute *t*-statistic of the individual-level regression weights in the hierarchical Bayesian linear regression model for each of the predictor variables in a 4-day history. This shows that self-reported mood is the most important variable for predicting mood the next day, which is not surprising considering mood has a strong autocorrelation (Figure 4).

**Table 2:** Predictor variables sorted by overall feature importance measured by the mean absolute t-statistic of the individual-level regression parameters in the hierarchical Bayesian linear regression model for w=4 and h=1. Self-reported mood is the most important variable for predicting mood on the following day.

Predictor	|*t*|, mean (SD)			
	*x*_t_	*x*_t−1_	*x*_t−2_	*x*_t−3_
Mood	4.53 (3.35)	2.34 (0.55)	0.47 (0.28)	2.78 (0.18)
Anxiety	2.78 (0.05)	0.71 (0.02)	1.29 (0.01)	0.76 (0.00)
Irritable	2.74 (0.11)	1.22 (0.01)	0.95 (0.01)	1.30 (0.00)
Mixed mood	2.09 (0.06)	2.51 (0.02)	1.96 (0.01)	0.52 (0.01)
Medicine changed	0.36 (0.10)	0.08 (0.01)	2.15 (0.01)	0.64 (0.00)
Sleep positive	1.65 (0.01)	0.72 (0.00)	0.37 (0.00)	0.16 (0.00)
Cognitive difficulty	1.48 (0.09)	0.58 (0.02)	0.19 (0.00)	1.57 (0.00)
Alcohol	0.67 (0.02)	0.77 (0.01)	1.56 (0.01)	0.87 (0.00)
Medicine omitted	0.13 (0.01)	1.31 (0.00)	0.60 (0.00)	0.14 (0.00)
Stress	1.22 (0.12)	0.91 (0.02)	0.71 (0.01)	0.28 (0.01)
Activity	1.04 (0.03)	1.14 (0.02)	0.49 (0.01)	1.14 (0.01)
Sleep negative	0.41 (0.01)	0.52 (0.00)	0.48 (0.00)	0.52 (0.00)

### Time-Series Cross-Validation Results

The results of the leave-all-out and leave-one-out time-series cross-validation experiments for *w*=4 and *h*=1 are presented in Table 3. In both experiments the naïve pooled mean model scored a predicted *R*^2^ close to zero because it does not explain any variance in the data. A predicted *R*^2^ score greater than zero indicates that some variance is explained while a negative *R*^2^ score is worse than the pooled mean model. The *last observed* model simply repeats the last observed mood value, which performs considerably better than the mean model and represents a solid baseline.

**Table 3:** Results of the leave-all-out time-series cross-validation (left) and leave-one-out time-series cross-validation (right) experiments. The hierarchical Bayesian linear regression model achieves the best results. The pooled models are better than the separate models, overall.

Model	Leave-all-out		Leave-one-out	
	*R^2^* ^a^	RMSE^b^	*R^2^* ^a^	RMSE^b^
Last observed	0.342	0.376	0.151	0.385
Pooled mean	−0.007	0.465	−0.009	0.419
Pooled ridge	0.450	0.344	0.340	0.339
Pooled XGBoost	0.455	0.342	0.343	0.338
Separate mean	0.213	0.412	−0.443	0.502
Separate ridge	0.345	0.375	−0.471	0.506
Separate XGBoost	0.302	0.388	−0.682	0.541
Hierarchical Bayesian linear	0.511	0.324	0.347	0.337
Hierarchical Bayesian ordinal	0.495	0.330	0.343	0.339

^a^Coefficient of determination (*R^2^*): higher is better.

^b^Root mean squared error (RMSE): lower is better.

The leave-all-out time-series cross-validation experiment was evaluated with *T*=24 and data partitions a size of one week, resulting in *T*−1=23 iterations of cross-validation. The hierarchical Bayesian linear model achieved the best result with the predicted *R*^2^=0.511 and predicted RMSE=0.324, beating the naïve baseline and pooled and separate regression models. The hierarchical Bayesian ordinal model is a close second best.

The leave-one-out time-series cross-validation experiment was evaluated for each individual with the first 2 weeks of data pooled with data from the rest of the population in the training set and evaluated on the next 22 weeks of data from that individual, resulting in *J*=58 iterations of cross-validation. The hierarchical Bayesian linear model achieved the best predicted *R*^2^=0.347 and predicted RMSE=0.337, but is similar to the best pooled regression models, indicating that the hierarchical model does a lot of pooling as well. The separate models fail to generalize to the held-out test data in this experiment, achieving negative *R*^2^ scores, because the training sets contain only 2 weeks of data. Overall, the hierarchical and pooled models performed better than the separate models, and all regression models generally outperformed the naïve baseline models when sufficient data were available.

### Seven-Day Forecast

**Figure 6:**
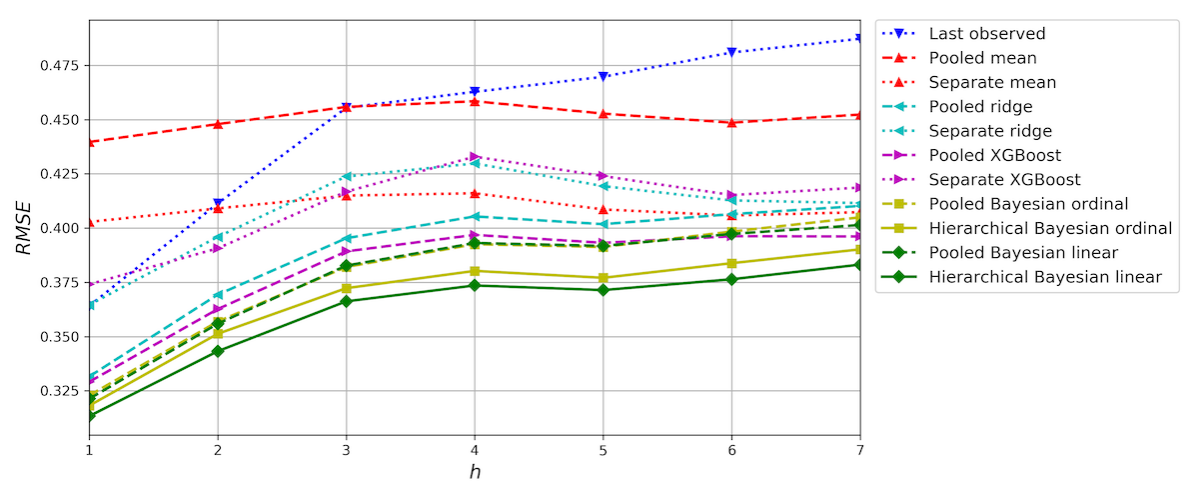
Results of forecasting mood for up to seven days. The root mean squared error (RMSE) was evaluated in time-series cross-validation experiments for w=4 and h=1 through 7. As expected, the RMSE increases when forecasting further ahead. The proposed hierarchical models achieved consistently lower RMSEs than the baseline models.

**Figure 7:**
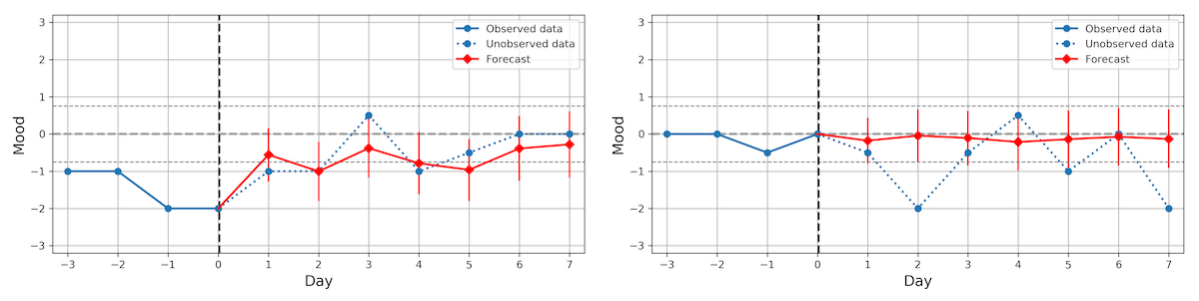
Examples of 7-day mood forecasts produced by the hierarchical linear regression model. The forecasted mood values are shown with 95% CI uncertainties and compared with observed data. The forecast to the left is rather accurate despite variation in the data, whereas the forecast to the right fails to anticipate future mood changes.

Thus far we have focused on evaluating a 1-day forecast, but it is also interesting to forecast mood on a more distant horizon. Figure 6 shows the mean RMSE of cross-validation for *w*=4 and *h*=1 through 7. The hierarchical Bayesian linear regression model achieves the lowest RMSE of all models for every value of *h*. As might be expected, the error generally grows with the size of the horizon. The errors of the naïve mean models are almost constant, varying only because of the difference in datasets available for different values of *h*. However, even at *h*=7, the best regression models are able to outperform the mean models, meaning they are able to capture useful information from prior self-assessments. Two examples of 7-day mood forecasts produced by the hierarchical linear regression model are presented in Figure 7.

## Discussion

### Principal Findings

In this study, we have analyzed smartphone-based self-assessment data from a population of 84 patients with bipolar disorder with the purpose of forecasting subjective mood. The initial data analysis showed that the majority of observed mood scores are close to zero, indicating weak or no symptoms among the population for most of the study period. Yet, we found a significant negative correlation between self-reported mood scores and HDRS scores (*r*=−0.40; *P*<.001) and a significant positive correlation between self-reported mood scores and YMRS scores (*r*=0.22; *P*<.001). This confirms prior findings [40-42], suggesting that subjective mood is a valid indicator of the mental state in bipolar disorder and thereby also a clinically relevant feature for daily monitoring and forecasting. We did not observe any substantial seasonality or long-term trend of subjective mood, indicating that time-series models designed to utilize such time-dependent patterns [32] are not appropriate for forecasting mood. However, the recorded mood scores do show an autocorrelation several days ahead. Thus, we employed a multiple regression approach based on a history of predictor variables to forecast future mood scores. In particular, we proposed using a hierarchical Bayesian model to perform MTL, enabling personalized predictions while considering common characteristics of the population. The hierarchical approach additionally makes it possible to reason about individuals for whom we have observed little data, thus overcoming the cold start problem.

Employing a regression model approach to produce a forecast required us to find an appropriate window size defining the predictor variables included in the model. With perfect data and a model robust to overfitting, increasing the window size should never result in a worse model, as any added noninformative variables could simply be ignored. In a real-world application, however, increasing the window size often results in fewer training examples because of missing data and similarly requires more data to enable prediction on new instances. Thus, finding the optimal window size is a trade-off that depends on data quality and model robustness. In our experiment, we found that including a history of up to four days improved the prediction error, but with more complete data, there is no reason the window size could not be increased even further. For instance, Suhara et al [22] found that their model for classifying depression benefited from long data histories up to 14 days, although it is our experience that collecting complete self-assessment histories over an extended period is very difficult.

By inspecting the inferred regression parameters of the hierarchical Bayesian model, we found historical mood to be the most important predictor of future mood. This result is not surprising as substantial changes in mood often occur over several days, and thus, future mood is likely to be similar to the mood in the immediate past. Consequently, the forecast is inclined to extrapolate the mood from previous days and gradually regress toward the mean of the data as uncertainty grows when forecasting further ahead. Although this forecast behavior succeeds at achieving a low error, its utility in a practical monitoring setting must be studied further. We see this as an interesting topic for future research. However, the results presented in this paper show that regression models based on self-assessment histories are able to consistently outperform naïve forecast baselines of either repeating the last observed value or predicting the mean of the pooled or separate data distributions up to seven days into the future (see Figure 7).

The proposed hierarchical linear and ordinal models achieved the best predictive performance in the time-series cross-validation experiments. In the leave-all-out cross-validation, the hierarchical Bayesian linear regression model achieved the best result (*R*^2^=0.511; RMSE=0.324) with the hierarchical Bayesian ordinal model being a close second. In the leave-one-out cross-validation, the hierarchical Bayesian linear regression model also achieved the best result (*R*^2^=0.547; RMSE=0.337) but was much closer to the performance of the best pooled models. These results show how the hierarchical approach solves the cold start problem by including information from the population when little individual data are observed and by gradually becoming more personalized as more data become available. In contrast to previous work, we found that pooled models outperformed separate models, indicating that the individual datasets did not contain sufficient information to produce accurate forecasts. Thus, the separate models were biased and consequently it proved more useful to disregard individual differences and include data from the population in a general model. The hierarchical models succeeded in finding a compromise between the pooled and separate approach by regularizing the personalized models with data from the population.

In forecasting mood for several days, the hierarchical models similarly achieved the best results. As expected, the forecast error increased when forecasting further ahead; however, we observed that the best regression models performed better than the naïve mean models for up to seven days. It is a remarkable result that a short self-assessment history of just a few days can forecast mood for several days, the most important reason being that substantial mood changes often happen gradually over a horizon longer than 7 days.

The data analyzed in this study were collected from a population of well-characterized patients with bipolar disorder during the MONARCA II randomized clinical trial [27] conducted by researchers with specific knowledge of bipolar disorder. Overall, the findings from this study are found to be generalizable to patients with bipolar disorder not presenting with an acute affective episode and who are willing to use a smartphone-based monitoring tool.

### Limitations

We observed a low prevalence of severe symptoms in our data sample leading to some limitations. As the mood values have low variance, regression models will tend to regress toward the mean of the data, and naïve mean models are able to achieve low errors relative to the full range of the mood scale. It prevented us from assessing how well the proposed method performs in a population with more severe symptoms and how well the forecast is at anticipating severe cases.

A major motivation for our research and the MONARCA II study was to establish a real-time mood forecasting solution to improve monitoring and enable early intervention in patients with bipolar disorder [27]. However, it is still not clear how a real-time forecast system is affected by interventions, as the intervention can change the outcome and thus future training data, which could lead to a biased model that underestimates future mood scores. Thus, it would be crucial to monitor the performance of a real-time system continuously using held out, unbiased data for validation.

### Perspectives

The mood forecast presented in this paper has used a history of self-reported features as input. However, several research projects have been investigating the use of sensor-based and automatically collected data as input for mood prediction. Sensor technology in modern smartphones enables tracking of a variety of behavioral features such as physical activity, location, and sleep along with communication and device usage logs. Additionally, sensor data can be captured with wearables such as wristbands and fitness trackers with high accuracy. Such sensor-based features could be used to augment or even reduce self-assessment in mood prediction tasks and thus reduce the need to prompt users for daily self-assessments. There is great potential in utilizing objectively collected sensor data in semiautomatic mood detection and forecasting.

Mood prediction and forecasting can be used as early warning signs in clinical treatment. Furthermore, accurate symptom forecasting could be extended to detect risk of relapse of major affective episodes specifically, eg, by detecting if values exceed predefined thresholds over consecutive days. This could be useful in, eg, a telemedicine setup in which trained nurses or other clinical personnel supervise patients in outpatient treatment. This could help catch early onset of major depressive or manic phases that can be addressed and handled early, which again could reduce the severity of symptoms and the degree of treatment. Hence, the need for readmission could be reduced. We are currently working on implementing a Web-based forecasting system evaluated as part of the RADMIS (reducing the rate and duration of readmissions among patients with unipolar disorder and bipolar disorder using smartphone-based monitoring and treatment) trials [43] to study its practical application, including investigating if such a system could potentially reduce readmission and hospitalization.

In this paper, we have examined the technical foundation of mood forecasting aimed at improving continuous disease monitoring. However, for a patient, the prospect of experiencing depressed or elevated mood in the future might lead to changes in behavior and state of mind and, in the worst case, become a self-fulfilling prophecy. Therefore, real-time mood forecasting should be used with care and applied exclusively as a monitoring and early intervention tool for professionals rather than being presented directly to users.

### Conclusions

Continuous symptom monitoring and early detection are important components in the treatment of patients with bipolar disorder. Smartphones provide a unique platform for self-assessment and management of depression and mania and have the additional benefit of making data available for immediate analysis. In this work, we have examined the feasibility of establishing a mood forecast system based on self-assessments to provide additional insights and enable early intervention. We found that our proposed method of applying hierarchical Bayesian regression models was able to consistently outperform commonly used machine learning methods and forecast subjective mood for up to seven days.

## Appendix

Multimedia Appendix 1 Stan code of the hierarchical linear regression and ordinal regression models and details on choice of model priors.

## Abbreviations

EMA: ecological momentary assessment
HDRS: Hamilton Depression Rating Scale
MTL: multi-task learning
RADMIS: reducing the rate and duration of readmissions among patients with unipolar disorder and bipolar disorder using smartphone-based monitoring and treatment
RMSE: root mean squared error
YMRS: Young Mania Rating Scale
