# Supplementary material for: Forecasting Mood in Bipolar Disorder From Smartphone Self-assessments: Hierarchical Bayesian Approach
Source: JMIR Mhealth Uhealth. 2020 Apr 1;8(4):e15028. doi: 10.2196/15028 (PMC7367518; doi:10.2196/15028)
Supplement: Multimedia Appendix 1 [file mhealth_v8i4e15028_app1.pdf]

Multimedia Appendix 1: Stan code

This multimedia appendix includes the Stan code of the hierarchical linear regression and ordinal regression models (see below). In practice, the Stan code was wrapped in Python classes and used a string Template to customize the code. In particular, the numerical parameters of the hyperpriors were adjustable and the generated quantities section could be left out for increased sampling speed when it was not required. The Python classes additionally provided a scikit-learn style interface for for fitting the model and making predictions as well as automatic caching of the compiled Stan model.

Priors

Because of our prior believe that current and previous observations of mood would be the strongest predictor of future mood, the population parameters of the hierarchical models corresponding to mood were assigned wider priors and the other population parameters were assigned narrower, more restrictive priors. In the hierarchical linear regression model, mean parameters, μ, corresponding to mood were assigned $\text{Normal}(0, 1)$ priors and population variance parameters, τ, were assigned $\text{Normal}(0, 0.1)$ priors. Mean parameters, μ, not corresponding to mood were assigned $\text{Normal}(0, 0.1)$ priors and population variance parameters, τ, were assigned $\text{Normal}(0, 0.01)$ priors. In the hierarchical ordinal regression model, mean parameters, μ, corresponding to mood were assigned $\text{Normal}(0, 10)$ priors and population variance parameters, τ, were assigned $\text{Normal}(0, 1)$ priors. Mean parameters, μ, not corresponding to mood were assigned $\text{Normal}(0, 1)$ priors and population variance parameters, τ, were assigned $\text{Normal}(0, 0.1)$ priors.

Additionally, our prior believe was that observed variables closer in time to the predicted future mood were more important, thus each prior was scaled with 1/lag of the corresponding predictor variable, i.e. for window size 4 ($w = 4$), population priors corresponding to predictor variables from the current day were scaled with 1/1, priors corresponding to the previous day were scaled with 1/2, priors corresponding to two days ago were scaled with 1/3 and priors corresponding to three days ago were scaled with 1/4. This helped to further regularize the model.

```
// Hierarchical linear regression model
data {
    int<lower=1> N;               // number of examples
    int<lower=1> D;               // number of predictors (dimensions)
    int<lower=1> J;               // number of groups
    int<lower=1,upper=J> tid[N];  // group identifier index vector
    row_vector[D] X[N];           // example vectors
    real y[N];                    // target vector
}
parameters {
    real mu_a;                    // intercept mean
    real<lower=0> tau_a;          // intercept deviation
    vector[D] mu_b;               // coefficient means
    vector<lower=0>[D] tau_b;     // coefficient deviations
    real alpha_raw[J];            // intercepts
    vector[D] beta_raw[J];        // coefficients
    real<lower=0> sigma;          // noise scale
}
transformed parameters {
    real alpha[J];
    vector[D] beta[J];
    for (j in 1:J) {
        alpha[j] = mu_a + tau_a * alpha_raw[j];
        beta[j] = mu_b + tau_b .* beta_raw[j];
    }
}
model {
    mu_a ~ normal(0, 1);
    tau_a ~ normal(0, 1);
    mu_b ~ normal(0, 1);
    tau_b ~ normal(0, 1);
    for (j in 1:J) {
        alpha_raw[j] ~ normal(0, 1); // normal(mu_a, tau_a)
        beta_raw[j] ~ normal(0, 1); // normal (mu_b, tau_b)
    }
    sigma ~ normal(0, 1);
    {
        vector[N] z;
        for (i in 1:N)
            z[i] = alpha[tid[i]] + X[i] * beta[tid[i]];
        y ~ normal(z, sigma);
    }
}
generated quantities {
    vector[N] log_lik;
    for (i in 1:N)
        log_lik[i] = normal_lpdf(y[i] | alpha[tid[i]] + X[i] * beta[tid[i]], sigma);
}
```

```
// Hierarchical ordinal regression model
data {
    int<lower=1> N;               // number of examples
    int<lower=1> D;               // number of predictors
    int<lower=2> K;               // number of classes
    int<lower=1> J;               // number of groups
    int<lower=1,upper=J> tid[N];  // group identifier index vector
    row_vector[D] X[N];           // examples
    int<lower=1, upper=K> y[N];   // targets
}
parameters {
    ordered[K-1] mu_c;            // cutpoint means
    real<lower=0> tau_c;          // cutpoint deviations
    ordered[K-1] c_raw[J];        // cutpoints
    vector[D] mu_b;               // coefficient means
    vector<lower=0>[D] tau_b;     // coefficient deviations
    vector[D] beta_raw[J];        // coefficients
}
transformed parameters {
    ordered[K-1] c[J];
    vector[D] beta[J];
    for (j in 1:J) {
        c[j][1] = mu_c[1] + tau_c * c_raw[j][1];
        for (k in 2:(K-1))
            c[j][k] = fmax(c[j][k-1] + 1.0e-5, mu_c[k] + tau_c * c_raw[j][k]);
        beta[j] = mu_b + tau_b .* beta_raw[j];
    }
}
model {
    mu_c ~ normal(0, 1);
    tau_c ~ normal(0, 1);
    mu_b ~ normal(0, 1);
    tau_b ~ normal(0, 1);
    for(j in 1:J) {
        c_raw[j] ~ normal(0, 1);
        beta_raw[j] ~ normal(0, 1); // normal(mu_b, tau_b);
    }
    for (i in 1:N)
        y[i] ~ ordered_logistic(X[i] * beta[tid[i]], c[tid[i]]);
}
generated quantities {
    vector[N] log_lik;
    for (i in 1:N)
        log_lik[i] = ordered_logistic_lpmf(y[i] | X[i] * beta[tid[i]], c[tid[i]]);
}
```
